# Severe Familial Chylomicronemia Syndrome With Refractory Hypertriglyceridemia and Multisystem Complications Managed With Chronic Plasmapheresis and Olezarsen: A Case Report

**DOI:** 10.7759/cureus.105594

**Published:** 2026-03-21

**Authors:** Christopher M Ahmad, Rae-Anne Kastle, Hima Patel, Samira R Haberman, Miriana Youkhana

**Affiliations:** 1 Medicine, Kansas City University, Joplin, USA; 2 Family Medicine, Rosalind Franklin University of Medicine and Science, Chicago, USA

**Keywords:** exocrine insufficiency, familial chylomicronemia syndrome, olezarsen, pancreatitis, plasmapheresis

## Abstract

Familial chylomicronemia syndrome (FCS) is a rare, inherited lipid disorder characterized by severe hypertriglyceridemia and a risk of recurrent pancreatitis. Patients with biallelic pathogenic variants affecting lipoprotein lipase (LPL)-mediated triglyceride metabolism may remain refractory to conventional lipid-lowering therapies and strict dietary control, leading to recurrent critical illness and progressive multisystem complications.

We report a female patient with genetically confirmed FCS and persistent triglyceride levels typically in the 4,000-5,000 mg/dL range despite adherence to diet and lipid-lowering therapy, without clear secondary contributors to severe hypertriglyceridemia. Her course included recurrent intensive care unit (ICU) admissions for hypertriglyceridemia-associated pancreatitis requiring insulin drips, with progression to chronic pancreatitis, pancreatic insufficiency requiring enzyme replacement, insulin-dependent diabetes with continuous glucose monitoring (CGM), chronic pain syndrome with opioid dependence concerns, and psychiatric comorbidity. During a hospitalization approximately 10 months prior to the most recent follow-up, the patient underwent placement of a right chest tunneled central venous catheter and initiated therapeutic plasmapheresis. She was concurrently followed by a triglyceride clinic and continued on olezarsen. At follow-up on February 25, 2025, she reported no hospitalizations since April 2024. A triglyceride value of 4,700 mg/dL was documented shortly before a scheduled plasmapheresis session.

This case highlights the complexity of severe FCS when hypertriglyceridemia remains refractory to conventional management and illustrates a care pathway in which chronic outpatient plasmapheresis combined with emerging RNA-based therapy was associated with stabilization and avoidance of recurrent hospitalization. Sustained outpatient success required multidisciplinary coordination, addressing pancreatitis sequelae, glycemic management, chronic pain, psychiatric disease, and central-line monitoring. These observations are hypothesis-generating and highlight the potential role of coordinated outpatient plasmapheresis and emerging RNA-based therapies in the management of severe FCS.

To our knowledge, reports describing long-term outpatient stabilization of severe FCS using combined chronic plasmapheresis and apolipoprotein C-III (APOC3)-targeted RNA therapy remain limited, and this case highlights a potential care pathway for patients with refractory disease.

## Introduction

Familial chylomicronemia syndrome (FCS) is an ultra-rare genetic disorder of triglyceride-rich lipoprotein metabolism, typically inherited in an autosomal recessive pattern, and associated with profound elevations in serum triglycerides [1-3]. FCS has an estimated prevalence of approximately 1-2 cases per 1,000,000 individuals worldwide [4]. Clinically, FCS is notable for recurrent episodes of pancreatitis, which may be severe and require intensive care. Recurrent episodes may lead to chronic pancreatitis, with exocrine and endocrine pancreatic failure over time [1]. In contrast to more common polygenic or secondary hypertriglyceridemia, monogenic chylomicronemia can remain markedly resistant to standard triglyceride-lowering approaches, including diet and conventional lipid medications, such as fibrates [1-3].

FCS is distinguished from more common multifactorial hypertriglyceridemia by its persistent severity, early clinical burden, and frequent refractoriness to standard triglyceride-lowering approaches. In affected individuals, triglyceride concentrations may remain profoundly elevated, despite strict dietary restriction and multiple lipid-lowering agents. These patients frequently experience recurrent episodes of pancreatitis, which, over time, may progress to chronic pancreatitis and subsequent pancreatic endocrine and exocrine dysfunction [1-3]. FCS results from biallelic pathogenic variants in genes involved in lipoprotein lipase (LPL)-mediated triglyceride metabolism, most commonly the LPL gene [5].

Recently, the U.S. Food and Drug Administration (FDA) approved olezarsen (Tryngolza), an antisense oligonucleotide targeting apolipoprotein C-III (APOC3), for the treatment of adults with FCS [6].

We describe a patient with confirmed FCS, no clear secondary contributors to hypertriglyceridemia, and recurrent intensive care unit (ICU) admissions for hypertriglyceridemia-associated pancreatitis, whose disease course evolved into multisystem chronic illness. We focus on escalation to chronic weekly plasmapheresis via a tunneled catheter, combined with ongoing olezarsen therapy, highlighting the transition from recurrent ICU-level illness to outpatient stabilization, and emphasizing the system-based care coordination required to sustain long-term management outside the hospital.

## Case presentation

Patient and baseline clinical course

The patient is a 43-year-old female with genetically confirmed FCS and longstanding severe hypertriglyceridemia. Clinical documentation describes persistently elevated triglyceride values in the 4,000-5,000 mg/dL range, despite adherence to dietary recommendations and lipid-lowering medications. Genetic testing was performed to evaluate for monogenic causes of severe hypertriglyceridemia. Results demonstrated two pathogenic variants in the LPL gene. Variant 1 was c.662T>C (p.Ile221Thr), and Variant 2 was c.835C>G (p.Leu279Val). The patient was therefore compound heterozygous for pathogenic variants in LPL, consistent with autosomal recessive FCS. Family history was notable for parental consanguinity (first-cousin relationship), which may increase the likelihood of autosomal recessive lipid disorders, such as FCS.

Secondary contributors to hypertriglyceridemia were evaluated and minimized; the patient reported no alcohol use, was a lifelong non-smoker, and her medication review did not identify agents commonly associated with severe hypertriglyceridemia (e.g., estrogens, corticosteroids, or atypical antipsychotics). She had a stable weight history, with a body mass index of 18.8 kg/m² (weight 93 lbs and height 4 ft 11 in), consistent with a lean body habitus without features of metabolic syndrome. The absence of identifiable secondary factors supported a primary monogenic etiology.

During an April 2024 hospitalization for severe hypertriglyceridemia and pancreatitis, the patient underwent placement of a tunneled, right-sided central venous catheter and initiation of therapeutic plasmapheresis. Plasmapheresis was initially performed weekly in the outpatient setting and was later escalated to twice-weekly treatments due to persistently elevated triglyceride levels. This regimen was continued through a specialized infusion center as part of the patient’s ongoing outpatient management.

Prior to initiation of chronic outpatient plasmapheresis in April 2024, the patient experienced 12 hospitalizations for hypertriglyceridemia-associated pancreatitis between April 2023 and April 2024, including multiple ICU-level admissions requiring insulin infusion therapy. The frequency and severity of these admissions prompted escalation to extracorporeal lipid removal with chronic outpatient plasmapheresis. Following initiation of outpatient plasmapheresis, no additional hospitalizations for pancreatitis were documented through the most recent follow-up in February 2025.

Past medical history and problem list documented during follow-up included: hypertriglyceridemia/FCS; severe chronic pancreatitis related to hypertriglyceridemia; pancreatic insufficiency; insulin-dependent diabetes with hypoglycemia risk; chronic pain syndrome (including lumbar stenosis and herniated lumbar disc); migraine disorder; generalized anxiety disorder; major depressive disorder; chronic iron deficiency anemia secondary to blood loss; chronic angina; gastroesophageal reflux disease; hypotension; urinary incontinence; osteoporosis; vitamin D deficiency; and vitamin B12 deficiency.

**Figure 1:**
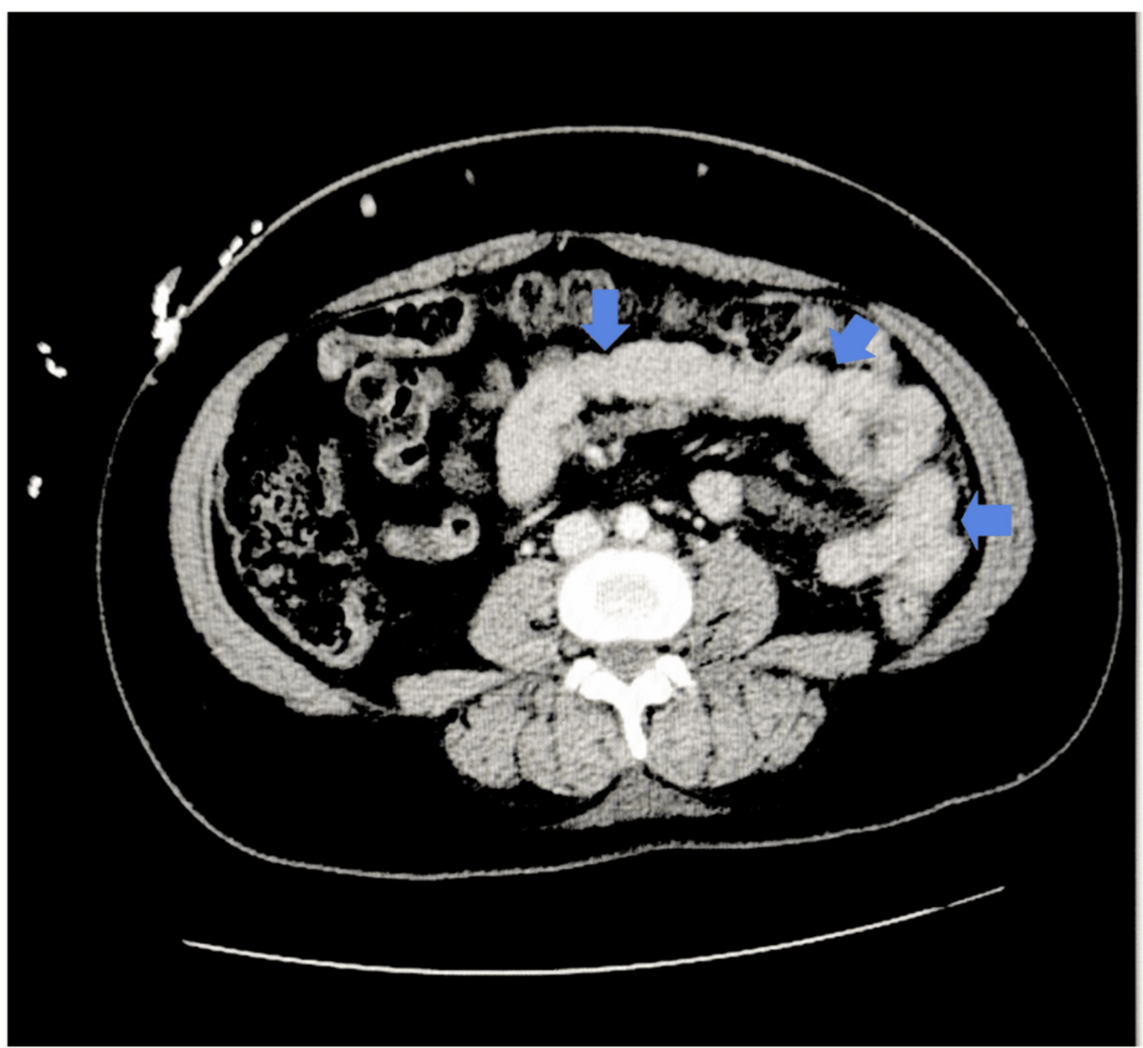
Axial CT image demonstrating peripancreatic fluid in acute pancreatitis. Axial contrast-enhanced CT image of the abdomen demonstrating inflammatory changes surrounding the pancreas. Peripancreatic fluid and inflammatory stranding are visible around the pancreatic body and tail (arrows), which are findings consistent with acute interstitial pancreatitis. CT: computed tomography

Contrast-enhanced computed tomography (CT) of the abdomen demonstrated inflammatory changes centered around the pancreas. Axial imaging revealed peripancreatic fluid and inflammatory stranding surrounding the pancreatic body and tail, consistent with acute interstitial pancreatitis (Figure 1).

**Figure 2:**
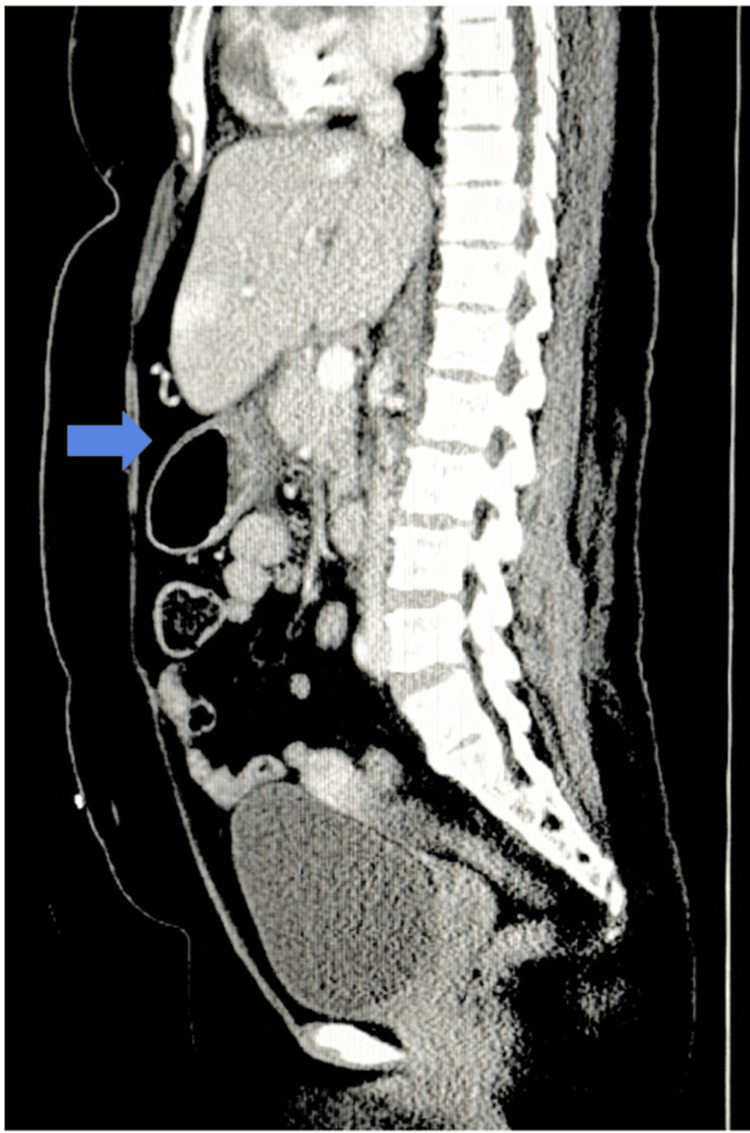
Sagittal CT reconstruction demonstrating peripancreatic inflammation adjacent to the stomach. Sagittal reconstruction demonstrating peripancreatic inflammatory fluid tracking adjacent to the greater curvature of the stomach, with associated gastric wall thickening (arrow, reflecting reactive inflammatory changes secondary to pancreatitis). CT: computed tomography

Sagittal reconstruction further illustrated inflammatory changes extending along the posterior aspect of the stomach, with associated gastric wall thickening, likely representing reactive inflammation secondary to adjacent pancreatic pathology (Figure 2).

The formal radiology interpretation confirmed moderate peripancreatic fluid accumulation surrounding the pancreatic body and tail, without evidence of necrosis, pseudocyst formation, or vascular compromise (Table 1). The imaging findings were consistent with acute interstitial pancreatitis, without radiographic evidence of pancreatic necrosis, consistent with a less severe morphologic classification based on available imaging.

**Table 1:** Radiologic findings from contrast-enhanced CT of the abdomen and pelvis. CT: computed tomography

Category	Radiologic Finding	Clinical Interpretation
Pancreas	Moderate peripancreatic fluid predominantly surrounding the pancreatic body and tail	Findings concerning for acute interstitial pancreatitis
Pancreatic complications	No pancreatic necrosis, no pseudocyst formation, portal vein and superior mesenteric veins patent	No evidence of severe pancreatitis complications
Stomach	Fluid adjacent to the greater curvature of the stomach with associated gastric wall thickening	Likely reactive inflammatory change secondary to pancreatitis; gastritis cannot be excluded
Adnexa	3.5 cm hypoattenuating lesion in the left adnexa	Concerning about ovarian cyst
Liver	Low-attenuation hepatic parenchyma with a focal area of higher density in the left hepatic lobe	Findings suggestive of hepatic steatosis with possible focal fat sparing or transient attenuation difference
Spleen	Prominent spleen not meeting criteria for splenomegaly	No significant splenic abnormality
Kidneys and adrenal glands	No suspicious renal mass or hydronephrosis; adrenal glands normal	No acute pathology
Bowel	No evidence of bowel obstruction	Normal bowel caliber
Surgical findings	Surgical suture material along the medial aspect of the cecum	Likely related to prior appendectomy
Free air or fluid	No intraperitoneal free air or free fluid	No evidence of perforation

Escalation to chronic plasmapheresis and outpatient infusion workflow

The patient’s clinical progression from recurrent pancreatitis episodes to a structured outpatient management strategy, incorporating chronic plasmapheresis and multidisciplinary care, is summarized in Table 2. Serial lipid measurements demonstrated substantial fluctuations in triglyceride levels, ranging from 244 mg/dL to 4,119 mg/dL, throughout the patient’s clinical course. Several measurements exceeded 1,000 mg/dL, a threshold strongly associated with increased risk of hypertriglyceridemia-induced pancreatitis. Longitudinal triglyceride measurements are summarized in Table 3, demonstrating persistently severe hypertriglyceridemia, both prior to and following initiation of outpatient plasmapheresis.

**Table 2:** Clinical evolution and longitudinal case timeline. This timeline illustrates the patient's transition from a cycle of acute medical crises to a stabilized, albeit high-intensity, outpatient management phase. The pivotal shift occurred in April 2024, with the move toward extracorporeal therapy (plasmapheresis). FCS: familial chylomicronemia syndrome; ICU: intensive care unit; PTSD: post-traumatic stress disorder; TG: triglyceride

Time Point	Clinical Event/Milestone	Outcome & Significance
Childhood-Adulthood	Diagnosis of FCS	Establishment of genetic metabolic baseline; history of extreme hypertriglyceridemia
2020-2023	Recurrent acute pancreatitis with multiple ICU admissions	Cumulative damage to pancreatic tissue; onset of chronic pain and endocrine dysfunction
December 2023	Critical ICU admission for severe acute pancreatitis	Catalyzed the transition from conservative management to aggressive interventional therapy
April 2024	Tunneled catheter placement; initiation of weekly plasmapheresis	Introduction of mechanical lipid removal to stabilize triglyceride levels and prevent recurrence
May-July 2024	Psychiatry and pain management referrals initiated	Transition to a multidisciplinary approach addressing "medical PTSD" and chronic pain syndrome
February 2025	Last documented triglycerides 4,700 mg/dL	Despite high TG levels, the absence of hospitalizations suggests successful stabilization via current management

**Table 3:** Longitudinal triglyceride measurements during the patient’s clinical course with key clinical events.

Date	Triglycerides (mg/dL)	Clinical Context/Annotation
April 29, 2024	861	Elevated triglycerides noted on lipid panel during outpatient evaluation
April 30, 2024	547	Persistent hypertriglyceridemia during hospitalization
May 14, 2024	4,119	Severe hypertriglyceridemia associated with acute pancreatitis episode
May 28, 2024	1,277	Partial improvement following inpatient management
June 4, 2024	1,741	Rebound elevation despite ongoing therapy
June 18, 2024	3,861	Recurrent severe hypertriglyceridemia consistent with refractory disease
August 23, 2024	1,518	Persistently elevated triglycerides during outpatient monitoring
September 10, 2024	1,837	Continued severe hypertriglyceridemia despite medical therapy
September 13, 2024	244	Marked reduction following therapeutic intervention (e.g., plasmapheresis)
September 20, 2024	1,431	Rebound elevation after initial triglyceride reduction

Prior to initiation of chronic plasmapheresis, the patient experienced 12 hospitalizations for pancreatitis over a 12-month period (April 2023-April 2024), corresponding to an approximate rate of one hospitalization per month. Following initiation of outpatient plasmapheresis in April 2024, no hospitalizations were documented over a 10-month follow-up period, resulting in zero hospitalizations per year during this interval.

Although complete pre- and post-plasmapheresis triglyceride pairs were not consistently available, available measurements demonstrate substantial variability, with intermittent reductions following therapeutic intervention. For example, triglyceride levels decreased from 1,837 mg/dL to 244 mg/dL within a three-day interval in September 2024, representing an approximate 86.7% reduction, consistent with an acute, treatment-associated reduction in triglyceride burden. However, subsequent rebound elevations (e.g., 244 mg/dL to 1,431 mg/dL within one week) highlight the persistent underlying defect in triglyceride metabolism, characteristic of FCS.

A representative lipid panel obtained during hospitalization demonstrates severe hypertriglyceridemia, with markedly reduced high-density lipoprotein (HDL) cholesterol, consistent with the biochemical profile of chylomicronemia (Table 4).

**Table 4:** Lipid panel obtained during hospitalization (April 29, 2024). HDL: high-density lipoprotein

Test	Value
Total Cholesterol	109 mg/dL
Triglycerides	861 mg/dL
HDL Cholesterol	<20 mg/dL
Non-HDL Cholesterol	89 mg/dL
Cholesterol/HDL Ratio	5.4

At a routine outpatient follow-up, approximately 10 months after initiation of chronic plasmapheresis, the care team documented that the patient had been avoiding hospitalization since April 2024. Notes indicated she had recently undergone plasmapheresis following a triglyceride measurement of 4,700 mg/dL, obtained prior to a scheduled session. Central line monitoring was explicitly documented, including a tunneled catheter site without erythema, swelling, or drainage; a clean, dry, intact dressing; and no signs of infection around the tunneled catheter.

Since initiation in April 2024, the patient has undergone numerous outpatient therapeutic plasmapheresis sessions. Treatments were initially performed weekly and were later intensified to twice-weekly sessions due to persistently elevated triglyceride levels.

Olezarsen therapy and lipid management context

The patient was initiated on olezarsen under a non-emergency Expanded Access protocol, approved by WCG IRB (Protocol: Olezarsen expanded access for FCS, IRB tracking number 20244259). This pathway was pursued due to persistent, severe hypertriglyceridemia and recurrent pancreatitis, despite maximal conventional therapy. Following subsequent formal regulatory approval of olezarsen, treatment was continued under standard prescribing protocols through a dedicated triglyceride clinic.

Conventional lipid-lowering therapy remained part of her regimen, including documented use of agents such as fenofibrate, gemfibrozil, ezetimibe, and omega-3 products, and she also had entries consistent with rosuvastatin and PCSK9 therapy (e.g., evolocumab/Repatha), reflecting extensive lipid-directed polypharmacy. Medication lists were reviewed and reconciled at the February 25, 2025, visit.

Gastrointestinal and pancreatic sequelae

The patient’s history included chronic pancreatitis, described as having multiple acute attacks over prior months and becoming more constant. She was managed for pancreatic insufficiency with pancreatic enzyme replacement therapy (Creon). Documentation referenced dietary counseling and monitoring for symptoms consistent with malabsorption. The abdominal exam at follow-up was described as soft, distended, and tympanitic to percussion, without rebound or guarding.

Endocrine complications and diabetes management

The patient carried a diagnosis of insulin-dependent diabetes, with documented hypoglycemia risk. Her diabetes regimen included basal and prandial insulin (documented entries for Tresiba and Humalog/lispro) and continuous glucose monitoring (CGM) using Dexcom G7. Emergency hypoglycemia rescue therapy (glucagon products) was listed. The chart narrative connected insulin dependence to pancreatic disease and hypertriglyceridemia-related complications, in the context of chronic pancreatitis. Glycemic control was monitored throughout the patient’s clinical course. Hemoglobin A1c measured 5.5% in January 2024, prior to the April hospitalization. Following the period of intensive insulin therapy required during hospitalization, HbA1c was measured at 5.2%. During outpatient follow-up in July 2024, HbA1c measured 5.6%. This suggests adequate management of pancreatogenic diabetes, despite significant metabolic instability and hypoglycemia risk.

Chronic pain and functional limitation

The patient had a long-standing history of lumbar stenosis, with chronic back pain rated 7-8/10, aggravated by walking and prolonged standing, and sometimes radiating down the legs, causing functional limitation. She had been on chronic opioid therapy (hydrocodone/acetaminophen listed), with documented challenges tapering due to worsening pain. The February 25, 2025, record included explicit documentation of a transition plan to pain management specialists, aligned with opioid-prescribing best practices and continuity planning.

Psychiatric comorbidity

The patient had documented major depressive disorder and generalized anxiety disorder. At follow-up, she reported persistent depressive symptoms, despite adherence to antidepressant therapy, including poor sleep, decreased appetite, fatigue, anhedonia, and impaired concentration; she denied suicidal ideation. Continued psychiatric follow-up and medication management were documented.

Objective findings and vitals

At the most recent outpatient evaluation, vital signs included a weight of 113 lb, a height of 58 in, a BMI of 23.61 kg/m², a temperature of 97.1°F, a blood pressure of 100/70 mm Hg, a heart rate of 75 bpm, and an oxygen saturation of 99%. General appearance was described as frail/thin habitus, alert, and oriented, without acute distress. Cardiopulmonary examination showed a regular rate and rhythm, without murmurs, and clear lung fields, without respiratory distress. Neurologic exam documented intact cranial nerves and no focal deficits. Musculoskeletal exam noted limited range of motion and an antalgic gait.

Care coordination and disability planning

The patient’s outpatient management required coordination across weekly plasmapheresis sessions, triglyceride clinic follow-up for olezarsen, diabetes monitoring and supplies, chronic pancreatitis management, psychiatric services, and referral for pain management (Table 5). Given the chronicity, multisystem burden, polypharmacy, and infusion dependence, documentation indicated a recommendation to apply for disability.

**Table 5:** Baseline clinical profile and multidisciplinary management strategy. A comprehensive clinical profile of a patient with FCS demonstrating the intersection of extreme hypertriglyceridemia, pancreatogenic diabetes, and high-intensity care requirements. FCS: familial chylomicronemia syndrome; TG: triglyceride; EPI: exocrine pancreatic insufficiency; CGM: continuous glucose monitor; GI: gastrointestinal; MSK: musculoskeletal

Clinical Domain	Key Problems & Pathologies	Ongoing Management (Status 2026)
Lipid	FCS; TG 4,000-5,000 mg/dL; refractory clinical course	Olezarsen (Tryngolza); weekly plasmapheresis; combination therapy with multiple lipid-lowering agents
Pancreas/GI	Chronic pancreatitis; secondary EPI	Creon (pancrelipase); gastrointestinal symptom monitoring; intensive dietary counseling (low-fat)
Endocrine	Insulin-dependent diabetes mellitus (Type 3c); significant risk for severe hypoglycemia	Tresiba (basal) + Humalog (prandial); Dexcom G7 CGM; emergency rescue glucagon kit
Pain/MSK	Lumbar stenosis; herniated disc; chronic pain syndrome; high risk for opioid dependence	Pregabalin and non-opioid adjuncts; established pain management referral and transition plan
Psychiatric	Major depressive disorder; generalized anxiety disorder	Dual pharmacotherapy (sertraline and duloxetine); regular psychiatry follow-up
Functional	Frail habitus; limited mobility; long-term infusion/treatment dependence	Long-term disability planning; integrated care coordination
Social	High burden of care; infusion/apheresis dependency	Weekly medical coordination; patient adherence monitoring

## Discussion

This case illustrates severe FCS as a chronic, multisystem disease, characterized by recurrent critical illness and progressive morbidity when triglyceride levels remain profoundly elevated, despite standard approaches. The patient’s course demonstrates persistent triglyceride values in the 4,000-5,000 mg/dL range, recurrent ICU admissions for pancreatitis requiring insulin drips, and the later development of chronic pancreatitis. 

Severe hypertriglyceridemia is a well-researched risk factor for acute pancreatitis. The risk rises significantly with triglyceride levels exceeding 1,000-2,000 mg/dL. The mechanism underlying pancreatic damage from triglycerides is pancreatic capillary ischemia, resulting from triglyceride-induced increases in chylomicron production and lipolytic free fatty acid release [7]. Chronic hypertriglyceridemia, leading to recurrent pancreatitis, is a recognized risk factor for pancreatogenic diabetes. Pancreatogenic diabetes, known as type 3c, is diabetes secondary to pancreatic damage. The development of type 3c diabetes is attributed to islet cell destruction, leading to insulin deficiency and impaired glucagon secretion, thereby increasing glycemic instability [8]. This patient demonstrated both exocrine insufficiency, requiring enzyme replacement, and insulin-dependent diabetes, consistent with the downstream consequences of sustained pancreatic injury. 

A key feature of this case is the transition from recurrent inpatient utilization to a structured outpatient maintenance strategy, centered on weekly plasmapheresis. Plasmapheresis is often considered in the setting of severe hypertriglyceridemia and pancreatitis risk; however, chronic maintenance use is less commonly described in routine outpatient care pathways [9]. In this patient, outpatient success depended on reliable vascular access, ongoing catheter-site surveillance, adherence to infusion center protocols, and careful care coordination. Importantly, objective follow-up documentation noted the absence of signs of catheter infection and intact dressings, supporting the feasibility of ongoing therapy when monitoring is consistent. Chronic therapeutic plasmapheresis also carries procedural risks, including catheter-related infection, thrombosis, bleeding, and electrolyte disturbances. However, large clinical series suggest that serious complications remain relatively uncommon when procedures are performed in experienced centers [10]. Although therapeutic plasma exchange is traditionally used for the acute management of hypertriglyceridemic pancreatitis, this case suggests that structured outpatient maintenance therapy may represent a viable strategy for selected patients with refractory FCS, when combined with disease-targeted pharmacologic therapy and coordinated multidisciplinary follow-up.

In parallel, the patient continued olezarsen under a triglyceride clinic, reflecting the incorporation of an emerging RNA-based approach to the management of severe hypertriglyceridemia [11,12]. APOC3 is located on triglyceride-rich lipoproteins and can inhibit LPL, block hepatic uptake, and promote inflammation, contributing to hypertriglyceridemia [13]. APOC3 inhibition reduced hepatic APOC3 production, thereby enhancing LPL-mediated clearance of triglyceride-rich lipoproteins [14]. Clinical studies of APOC3-targeted therapies, including a recent phase 3 randomized trial of olezarsen, have demonstrated significant reductions in triglycerides in patients with FCS [6,15]. In that study, the 80-mg dose produced a 43.5%-point greater reduction in triglyceride levels, compared with placebo at six months, and episodes of acute pancreatitis occurred far less frequently in the olezarsen groups than in the placebo group [6,16]. Olezarsen is a hepatocyte-targeted antisense oligonucleotide that reduces hepatic production of APOC3, thereby lowering circulating triglyceride levels and improving clearance of triglyceride-rich lipoproteins [16,17].

While triglyceride values remained markedly elevated before scheduled plasmapheresis sessions (e.g., 4,700 mg/dL shortly before treatment), the primary clinical endpoint observed in this case was the absence of hospitalization for pancreatitis since April 2024, suggesting that outcomes beyond triglyceride levels - such as admission frequency, pancreatitis recurrence, symptom stabilization, and functional status - may be important when evaluating therapeutic impact in severe FCS [1-3].

Although formal pancreatitis severity scores (e.g., BISAP or CTSI) were not consistently documented in the available records, the need for multiple ICU admissions, requiring insulin infusion, suggests episodes of at least moderate-to-severe disease.

**Figure 3:**
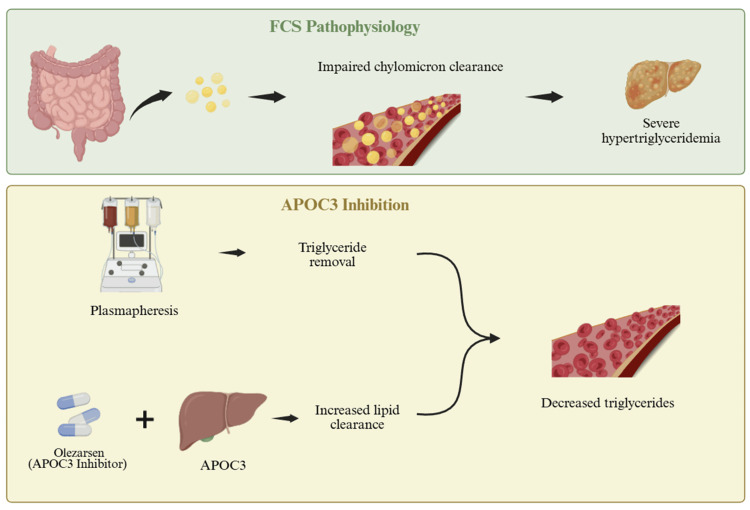
Conceptual overview of FCS and APOC3-targeted therapy. This author-created schematic illustrates impaired chylomicron clearance in FCS, the inhibitory role of APOC3 in triglyceride metabolism, and the therapeutic rationale for APOC3-targeted RNA therapy (olezarsen), with procedural triglyceride removal via plasmapheresis. The arrows indicate the direction of pathophysiologic processes and therapeutic effect. This figure is an original, author-created schematic using BioRender (Science Suite Inc., Toronto, Canada) and was not generated using AI. FCS: familial chylomicronemia syndrome; APOC3: apolipoprotein C3

The need to pursue olezarsen through a formal Expanded Access pathway underscores the limited therapeutic options historically available for patients with monogenic chylomicronemia, who remain refractory to dietary restriction and conventional lipid-lowering agents. Expanded Access programs are typically reserved for serious or life-threatening conditions, for which satisfactory alternatives are lacking. In this context, the patient’s disease severity and recurrent ICU admissions justified early access to APOC3-targeted RNA therapy, prior to full regulatory approval (Figure 3).

The case also highlights the importance of multidisciplinary management. Chronic pancreatitis drove endocrine and exocrine complications, requiring insulin, CGM, rescue glucagon availability, and pancreatic enzyme supplementation. Chronic pain and mobility limitations required structured opioid-risk mitigation and pain specialty transition planning. Psychiatric disease was persistent and clinically significant, with documented impact on sleep, appetite, and daily function, requiring ongoing pharmacologic management and follow-up [18]. Finally, the patient’s extensive medication list and multiple comorbidities emphasize the operational burden of polypharmacy, monitoring requirements, and specialist coordination, culminating in disability planning as part of comprehensive care.

Limitations

This case represents a single-patient experience and cannot establish causality or generalizability. Although hospitalization frequency decreased after initiation of weekly plasmapheresis and olezarsen, the temporal association does not prove therapeutic efficacy, particularly given concurrent dietary adherence, psychiatric stabilization, and multidisciplinary care. Serial triglyceride trends, standardized pancreatitis severity indices, and validated patient-reported outcome measures were not consistently collected, limiting objective pre-post comparison. Additionally, the absence of standardized pre- and post-plasmapheresis triglyceride pairs limits assessment of treatment-specific effects. As such, conclusions remain hypothesis-generating, and the long-term risks, costs, and sustainability of chronic outpatient plasmapheresis were not formally evaluated.

## Conclusions

Severe FCS can lead to profound, refractory hypertriglyceridemia, recurrent ICU-level pancreatitis, and progressive multisystem complications. In this case, escalation to weekly outpatient plasmapheresis, combined with ongoing disease-targeted therapy, coincided with a period of outpatient stability and avoidance of hospitalization. Long-term management required coordinated multidisciplinary care, addressing chronic pancreatitis sequelae, insulin-dependent diabetes, chronic pain, psychiatric comorbidity, and central-line monitoring. These observations highlight the complexity of managing refractory FCS and underscore the importance of coordinated, systems-based care in rare metabolic disease. Future studies are needed to better define the role of maintenance plasmapheresis in the era of emerging RNA-based therapies for FCS.
